# Hyponatremia as a prognostic factor for 30-day and 1-year mortality in patients acutely admitted to departments of internal medicine

**DOI:** 10.1186/1757-7241-23-S1-A18

**Published:** 2015-07-16

**Authors:** Louise Holland-Bill, Christian F Christiansen, Sinna P Ulrichsen, Troels Ring, Jens OL Jørgensen, Henrik T Sørensen

**Affiliations:** 1 Department of Clinical Epidemiology, Aarhus University Hospital, Aarhus, Denmark; 2 Department of Nephrology, Aalborg University Hospital, Aalborg, Denmark; 3 Department of Endocrinology and Internal Medicine, Aarhus University Hospital, Aarhus, Denmark

## Background

Little is known about the prevalence and prognostic impact of hyponatremia among patients admitted acutely to departments of internal medicine. We examined the prevalence of admission hyponatremia (serum sodium < 135 mmol/l) and its association with 30-day and 1-year mortality overall and according to diagnostic groups of previous morbidities and primary discharge diagnoses. Furthermore, we set out to identify threshold values predicting increased mortality, by treating serum sodium as a continuous variable.

## Methods

We used prospectively collected data from population-based registries to identify all first-time acute admission to departments of internal medicine in the Northern and Central Regions of Denmark from 2006 - 2011. We computed the prevalence of hyponatremia overall and for each hyponatremia category. Patients with hyponatremia and normonatremia were followed from the date of admission until death, migration, or up to one year. We used the Kaplan-Meier method (1 -- the survival function) to compute 30-day and 1-year mortality. Relative risks with 95% confidence intervals (CIs), adjusted for age group, gender, and previous morbidities, and stratified by clinical subgroups were estimated by the pseudo-value approach.

## Results

We identified 279,508 first-time acute admissions to departments of internal medicine in the study period. The prevalence of admission hyponatremia was 41,803 patients (15.0%), increasing with higher age and greater burden of previous morbidities. Thirty-day mortality was 3.6% in normonatremic patients compared to 7.3%, 10.0%, 10.4%, and 9.6% in patients with serum sodium levels of 130-134.9 mmol/l, 125-129.9 mmol/l, 120-124.9 mmol/l, and <120 mmol/l, resulting in adjusted relative risks (RRs) of 1.4 (95% CI: 1.3 to 1.4), 1.7 (95% CI: 1.6 to 1.8), 1.7 (95% CI: 1.4 to 1.9), and 1.3 (95% CI: 1.1 to 1.5), respectively. One-year mortality in patients with hyponatremia ranged from 20.2% to 24.8% compared to 10.6% in patients with normonatremia, with corresponding adjusted RRs of 1.3 (95% CI: 1.3 to 1.3), 1.4 (95% CI: 1.4 to 1.5), 1.4 (95% CI: 1.3 to 1.5), and 1.3 (95% CI: 1.1 to 1.4). Hyponatremia was associated with increased mortality risk across virtually all diagnostic groups. Sodium values of 132 mmol/l to 139mmol/l yielded the steepest increase in mortality.

## Conclusions

Admission hyponatremia, regardless of underlying morbidities, is associated with increased 30-day and 1-year mortality. The association with increased mortality seems independent of severity of hyponatremia.

